# Ein Vorschlag zur Reform der Slotvergabe an kapazitätsbeschränkten Verkehrsflughäfen

**DOI:** 10.1007/s10273-022-3216-2

**Published:** 2022-06-24

**Authors:** Andreas Knorr, Alexander Eisenkopf

**Affiliations:** 1 Verwaltungswissenschaften Speyer, Deutsche Universität für, Freiherr-vom-Stein-Straße 2, 67346 Speyer, Deutschland; 2 Zeppelin-LS f. Wirtschafts- u. Verkehrspolitik, Zeppelin Universität Friedrichshafen, Am Seemooser Horn 20 D, 88045 Friedrichshafen, Deutschland

**Keywords:** R41, R42, R48

## Abstract

Luftverkehrsdienste benötigen ergänzende boden- und luftseitige Infrastrukturen. Kapazitätsbeschränkungen des Start- und Landebahnsystems eines Flughafens stellen einen erheblichen Engpass dar. Das Nutzungsrecht hängt von der Verfügbarkeit eines Slots ab. Wenn die Nachfrage nach Slots das Angebot übersteigt, ist ein Zuweisungsverfahren erforderlich. In den meisten Ländern der Welt gibt es dafür keinen Marktmechanismus. Eine Besitzstandsklausel beschränkt den Zutritt für Neueinsteiger auf überlasteten Flughäfen. Aus der Perspektive der Eigentumsrechte lässt sich Abhilfe schaffen.

## Abstract (en)

Air transport services require complementary ground and air infrastructure. Capacity constraints of an airport’s runway system create a significant bottleneck. The right of use depends on the availability of a slot. When the demand for slots exceeds the supply, an allocation process is required. In most countries of the world, there is no market mechanism for this. A grandfather clause restricts entry for new entrants at congested airports. A remedy can be found in a property rights perspective.

Kaum eine Branche wurde von den weltweit zur Bekämpfung der Coronapandemie erlassenen staatlichen Restriktionen, insbesondere den Reisebeschränkungen und Quarantänevorschriften, so stark und nachhaltig getroffen wie die Luftverkehrswirtschaft (ICAO, 2022). So brachen die Passagierzahlen in den EU-Mitgliedstaaten 2020 gegenüber 2019 im Durchschnitt um 73,3 % ein (Eurostat, 2021). Zwar war von Mai bis Mitte Oktober 2021 wieder eine deutliche Verkehrszunahme zu verzeichnen, doch liegt das Aufkommen im Passagierverkehr nach wie vor deutlich unter dem Vorpandemiejahr 2019. So belief sich nach Angaben des Flughafenverbands ADV (2022) beispielsweise das Passagieraufkommen an den 22 größten deutschen Verkehrsflughäfen im November 2021 auf lediglich 49,5 % des monatlichen Vergleichswerts für 2019; der prozentuale Rückgang des Passagieraufkommens gegenüber dem jeweiligen Vergleichsmonat 2019 für den Zeitraum von Januar 2020 bis November 2021 ist in Abbildung 1 dargestellt.

**Prof. Dr. Dr. h. c. Andreas Knorr** ist Professor für Volkswirtschaftslehre, insbesondere für Wirtschafts- und Verkehrspolitik, an der Deutschen Universität für Verwaltungswissenschaften Speyer.

**Prof. Dr. Alexander Eisenkopf** hat den Zeppelin-Lehrstuhl für Wirtschafts- und Verkehrspolitik an der Zeppelin Universität Friedrichshafen inne.

Weit weniger betroffen war das Luftfrachtsegment, in dem das Niveau von 2019 bereits wieder übertroffen wurde und dessen Verkehrsleistung im weltweiten Mittel nun sogar einen historischen Höchststand erreicht hat (IATA, 2021). Anders als in der DACH-Region oder der EU hat sich das Passagieraufkommen in anderen Ländern inzwischen wieder spürbar erholt. So wurde im Dezember 2021 in den USA im Inlandsluftverkehr bereits wieder ein Wert von 85 % verzeichnet, und im grenzüberschreitenden Verkehr wurden 72 % des entsprechenden Aufkommens von 2019 erreicht (Airlines for America, 2022, 10). Aktuelle Prognosen gehen von einer vollständigen Erholung des Luftverkehrsmarkts nicht vor 2025 aus, bei beträchtlichen regionalen Unterschieden (Bain & Company, 2021).

Ein ähnlich massiver Einbruch wie bei den Passagierzahlen war auch bei der Zahl der gewerblichen Flugbewegungen, d. h. bei den Starts und Landungen im Passagier- und Frachtverkehr zu beobachten. Hier verzeichneten die deutschen Verkehrsflughäfen im April 2020 – dem verkehrsschwächsten Monat seit Ausbruch der Pandemie – einen Rückgang um 84,2 %. Im April 2021 hatte sich dieser Wert auf -59,8 % verbessert; im November 2021, dem letzten Monat, für den offizielle Daten vorliegen, betrug das Minus noch 29,1 % (Flughafenverband ADV, 2021, 5). Der im Vergleich zu den Passagierzahlen prozentual geringere Rückgang der Flugbewegungen spiegelt die deutlich niedrigere Auslastung der Flotten seit Pandemiebeginn wider. Die wirtschaftspolitische Diskussion in Deutschland und der EU zu den möglichen Auswirkungen der Coronapandemie auf die Luftverkehrsbranche beschränkte sich im Wesentlichen auf drei Problemfelder:

Abbildung 1

**Monatliches Passagieraufkommen an den deutschen Verkehrsflughäfen**

Veränderungsrate gegenüber dem jeweiligen Vergleichsmonat 2019

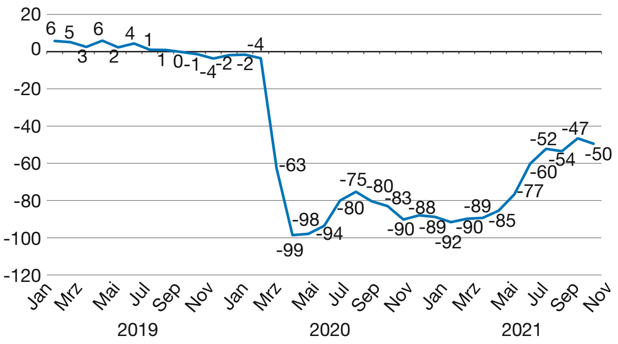

Quelle: Flughafenverband ADV (2022).

- Das (insbesondere) beihilferechtliche Pro und Contra staatlicher Rettungspakete zugunsten der wirtschaftlich stark angeschlagenen Fluggesellschaften und Flughäfen.
- Fragen des Verbraucherschutzes, vornehmlich hinsichtlich der Modalitäten der Kostenerstattung bei pandemiebedingten Flugannullierungen der Fluggesellschaften.
- Die Festlegung geeigneter und national wie international möglichst einheitlicher Hygienestandards als Voraussetzung für das Wiederhochfahren des Luftverkehrs.

Nur am Rande thematisiert wurde ein für die Wettbewerbsbedingungen auf den Luftverkehrsmärkten höchst relevanter Aspekt: Die Verfahren zur Allokation von Zeitnischen (Slots) zur Nutzung der Start- und Landebahnen an den hochfrequentierten und somit kapazitätsbeschränkten Verkehrsflughäfen. Deren allokative Ineffizienz ist seit langem weitgehend unbestritten (Grether, Isaac und Plott, 1981), zumal die Nichtverfügbarkeit von Slots für Newcomer eine kaum überwindliche Markteintrittsbarriere darstellt. Die Reformvorschläge beschränken sich auf unterschiedlich designte Auktionen (Bichler et al., 2021). Ihnen ist gemeinsam, dass sich die Analyse auf zwei Akteure beschränkt: den Staat, vertreten durch den jeweiligen nationalen Flugplankoordinator, als Anbieter knapper Slots und die Fluggesellschaften als deren Nachfrager. Demgegenüber werden die Flughäfen als Eigentümer der Start- und Landebahninfrastrukturen, deren Nutzungsrechte durch Slots alloziert werden müssen, außer Betracht gelassen. Aus einer De-lege-lata-Perspektive durchaus nachvollziehbar; jedoch lässt sich auch diskutieren, wie eine Grundsatzreform der Slotvergaberegeln einschließlich der Einbeziehung der Flughäfen als Akteure aussehen könnte.

## Kapazitätssteuerung an Verkehrsflughäfen

Beförderungsdienstleistungen im Luftverkehr benötigen komplementäre boden- und luftseitige Infrastrukturen (Knorr und Lueg-Arndt, 2018). Dabei umfasst die luftseitige Infrastruktur im weiteren Sinn den von der Flugsicherung bewirtschafteten Luftraum, innerhalb dessen sich ein Flugzeug vom Zeitpunkt des Abhebens bis zur Landung bewegt. Dazu komplementär ist die von den Flughäfen bereitgestellte luftseitige Infrastruktur im engeren Sinne, die sämtliche Bewegungsflächen für Flugzeuge am Flughafen, also das Start- und Landebahnsystem, die Rollwege (Taxiways) sowie die Parkpositionen am Terminal (Gates) oder auf dem Vorfeld (Apron) umfasst. Hinzu kommen die landseitigen Infrastrukturen des Flughafens.

Für die sichere und zugleich flugplankonforme Durchführung eines Fluges sind präzise aufeinander abgestimmte exklusive zeitliche Nutzungsrechte an luft- und bodenseitigen Infrastrukturen unabdingbar. Sie werden als Slots bezeichnet. Zu unterscheiden ist zwischen Flughafenslots (Airport Slots), die die Zahl der pro Stunde möglichen Starts- und Landungen widerspiegeln, und Luftstraßenslots (Airway Slots bzw. Calculated Take-off Time/CTOT). Unter der Annahme vorliegender Verkehrsrechte ist für die Durchführung eines Flugs somit die Zuteilung von drei komplementären Slots erforderlich: je ein Flughafenslot am Start- und Zielflughafen sowie ein Luftstraßenslot für die gewählte Flugstrecke. Hinzu kommt der zeitlich auf die Flughafenslots abgestimmte Zugang zu der für die Abfertigung des Flugs benötigten bodenseitigen Infrastruktur.

Die Kapazitätsauslastung ist je nach Flugstrecke, Tageszeit, Jahreszeit und Flughafen höchst unterschiedlich. Die Allokationsverfahren für Slots sind zudem nicht einheitlich. Luftstraßenslots kommen nur zum Einsatz, wenn die zuständigen Flugsicherungen ihre Kapazitätsgrenze erreicht haben, bis zu der die sichere horizontale und vertikale Staffelung der von ihr kontrollierten Flugzeuge im Luftraum noch gewährleistet werden kann. Erteilung und Inanspruchnahme erfolgen ausschließlich kurzfristig und tagesaktuell. Da Luftstraßen- gegenüber Flughafenslots generell Vorrang genießen, ist es etwa denkbar, dass ein verspätet erteilter Luftstraßenslot am Zielflughafen entweder keine Landung mehr erlaubt oder dort zur neuen verspäteten Ankunftszeit keine Flughafenslots mehr verfügbar sind.

Flughafenslots dienen der Disposition (Scheduling) der verfügbaren Start- und Landezeiten für alle gewerblichen und nichtgewerblichen Flüge an einem Verkehrsflughafen. Sie wurden Anfang der 1960er Jahre eingeführt, um der zunehmenden Verspätungshäufigkeit an hochfrequentierten Flughäfen entgegenzuwirken. Slots stellen seither eine zentrale Grundlage für die Flugplangestaltung der Fluggesellschaften dar. Sie werden von der rechtlich designierten nationalen Flugplankoordination eines Landes administriert. Das maximal verfügbare Angebot von Flughafenslots wird zum einen durch dessen Infrastrukturausstattung, insbesondere des Start- und Landebahnsystems, aber auch der Umschlagleistung aller bodenseitigen Abfertigungseinrichtungen, absolut begrenzt und als Koordinationseckwert des Flughafens bezeichnet (Fluko, 2022a). Diese werden ausschließlich für die als kapazitätsbeschränkt eingestuften Flughäfen festgelegt. Dabei werden internationalen Usancen gemäß zwei Kategorien unterschieden, wobei die Einstufung separat für jede der beiden halbjährigen Flugplanperioden erfolgt, d. h. für den Sommer- und den Winterflugplan (Fluko, 2022b und 2022c):

- An den flugplanvermittelten (*schedules facilitated*) Level-2-Flughäfen übersteigt die Nachfrage nach Slots das vorhandene Angebot lediglich temporär, also entweder an bestimmten Tageszeiten, an einzelnen Wochentagen oder saisonal. In Deutschland betrifft dies derzeit die Flughäfen Bremen, Dresden, Erfurt, Köln, Leipzig-Halle, Münster-Osnabrück, Nürnberg und Saarbrücken. Weltweit fielen in der Flugplanperiode Winter 2021 insgesamt 151 Verkehrsflughäfen in diese Kategorie (IATA, 2022a).

- An den koordinierten Level-3-Flughäfen besteht ein dauerhafter, erheblicher Nachfrageüberhang nach Flughafenslots. In Deutschland fallen die Flughäfen Berlin Brandenburg, Düsseldorf, Frankfurt Rhein/Main, Hannover, Hamburg, München und Stuttgart in diese Kategorie, die weltweit während der Flugplanperiode Winter 2021 insgesamt 170 Verkehrsflughäfen umfasste (IATA, 2022a).

Nach deutscher Rechtslage[1] obliegt die Zuständigkeit für die Festsetzung der Flughafenkapazität und des Koordinationseckwerts letztlich dem Bundesverkehrsministerium, das das Einvernehmen mit der obersten Luftfahrtbehörde des jeweiligen Bundeslands herzustellen hat; zuvor sind das Bundesaufsichtsamt für Flugsicherung, die Flugsicherungsorganisation, der Flughafenbetreiber sowie die Fluggesellschaften anzuhören. Mittelbar eingebunden ist auch der Koordinierungsausschuss des fraglichen Flughafens. Dieser setzt sich stets aus je einem Vertreter der Flugsicherung, des Flughafenbetreibers sowie der Spitzenverbände des gewerblichen Luftverkehrs und des Geschäftsreiseverkehrs zusammen. Darüber hinaus haben auch einzelne Luftfahrtunternehmen das Recht, je einen Vertreter in dieses Gremium zu entsenden. Die deutsche Flugplankoordination nimmt als Beobachterin an den Sitzungen des Koordinationsausschusses aller Flughäfen teil. Vergleichbare Regularien gelten in sämtlichen EU-Mitgliedstaaten und den meisten Staaten weltweit.

Wie unmittelbar einsichtig, ist das deutsche Verfahren zur Festsetzung der Koordinationseckwerte geeignet, Newcomer sowie alle Fluggesellschaften, die den Flughafen bislang nur unregelmäßig nutzen, mangels Repräsentanz zu benachteiligen. Demgegenüber werden die Koordinationseckwerte in Großbritannien, aber auch in Amsterdam Schiphol in Kooperation mit der Flugsicherung von den Flughafenbetreibern selbst festgelegt (Engemann, 2019).

## Verfahrensregeln der (Re-)Allokation von Flughafenslots

Infrastrukturengpässe an Verkehrsflughäfen wie eine Übernachfrage nach Flughafenslots stellen grundsätzlich eine Markteintrittsbarriere dar und schützen die bereits an diesem Standort aktiven Fluggesellschaften vor Newcomern. Ihr marktschließender Effekt hängt bei gegebener Kapazität folglich wesentlich vom Verfahren für die Zuteilung der knappen Slots auf die darum konkurrierenden Fluggesellschaften ab. Prozedural von höchster Relevanz sind in diesem Zusammenhang die „Worldwide Airport Slot Guidelines“ (WASG) der International Air Transport Association (IATA 2020). Sie ist der Branchenverband von derzeit 289 Fluggesellschaften aus 120 Ländern, auf die zusammen 83 % des Weltluftverkehrsaufkommens entfallen (IATA, 2022b).[2] Zwar hat die IATA seit der Öffnung der Luftverkehrsmärkte ab Ende der 1970er Jahre ihre Rolle als legales Preiskartell ihrer Mitglieder in wichtigen Verkehrsgebieten verloren. Große Bedeutung kommt ihr aber noch heute bei der Flugplankoordination zu, die auch die Verfahrensregeln zur Zuteilung von Flughafenslots beinhaltet. Die zuständigen IATA-Slotkonferenzen finden zweimal pro Jahr statt und dienen als „forum for the allocation of slots at fully coordinated airports (Level 3), and for the reaching of consensus on the schedule adjustments necessary to conform to airport capacity limitations (Level 2). The goal of the conference is for airlines and airports to obtain the slots that will give them the best possible schedule to offer their customers” (IATA, 2022b). Die IATA lehnt aus diesem Grund seit jeher jede Reform des bestehenden Systems grundsätzlich ab (IATA, 2017). Ihre WASG stellen rechtlich zwar nur unverbindliche Empfehlungen dar und sind in unterschiedlichem Maße rechtlich kodifiziert, werden aber gleichwohl weltweit umgesetzt. Gesetzliche Grundlage in der EU ist die seit ihrem Erlass mehrfach geänderte Verordnung (EWG) Nr. 95/93 des Rates vom 18. Januar 1993 über gemeinsame Regeln für die Zuweisung von Zeitnischen auf Flughäfen in der Gemeinschaft. Auch die USA folgen weitestgehend den IATA-Empfehlungen.

Den WASG gemäß erfolgt die Slotvergabe auf den nicht kapazitätsbeschränkten Flughäfen (Level 1) nach dem Wind-

1 § 27a des Luftverkehrsgesetzes in Verbindung mit der Verordnung über die Durchführung der Flughafenkoordinierung (FHKV) vom 13.6.1994. Die Bildung von Koordinierungsausschüssen und deren Zusammensetzung ist in § 2 der Verordnung geregelt.

2 Nahezu alle international tätigen Fluggesellschaften der Welt sind Mitglieder der IATA, während die große Mehrheit der weltweit führenden Low Cost Carrier ihr bislang nicht beigetreten sind.

hundverfahren (*first come, first served*). Für die flugplanvermittelten und koordinierten Flughäfen (Level 2 und Level 3) finden abgestufte Priorisierungsregeln Anwendung:

- Erste Priorität genießen die historischen Slots, also die einer Fluggesellschaft in der letzten relevanten Flugplanperiode bereits zugeteilten Slots. Diese Großvaterrechte werden automatisch verlängert. Allerdings besteht für sie eine 80 %ige Nutzungspflicht (Use-it-or-lose-it-Regel). Sie dürfen in Deutschland und der EU außerdem ohne Zustimmung der betroffenen Fluggesellschaft von der nationalen Flugplankoordination zeitlich nicht verlegt werden, selbst wenn dies in der Gesamtschau eine bessere Nutzung der knappen luftseitigen Infrastrukturkapazitäten des Flughafens erlauben würde.
- Zweite Priorität gilt für historische Slots, die von Fluggesellschaften konsensual und mit Zustimmung der Flugplankoordination auf ein anderes Zeitfenster verlegt wurden (*retimed slots*) oder zwischen Fluggesellschaften (auch innerhalb eines Konzernverbunds) unentgeltlich getauscht bzw. durch einen Unternehmenszusammenschluss übernommen wurden (Zeitnischenmobilität).
- Dritte Priorität genießen die in einem Slotpool zusammengefassten überzähligen Slots. Nach EU-Recht müssen diese in Zeitfenstern von je einer Stunde geordneten Slots hälftig an Neubewerbende vergeben werden, also an Fluggesellschaften, die im Sinne der Verordnung (EWG) 95/93 (Art. 2) am fraglichen Flughafen zuvor kaum aktiv waren. Der Slotpool speist sich zum einen aus neugeschaffenen Slots, z. B. durch die Eröffnung einer neuen Piste oder infolge neuartiger kapazitätserhöhender Flugsicherungsverfahren. Zum anderen speist er sich aus den Slots, die von den Fluggesellschaften freiwillig retourniert oder ihnen von der Flugplankoordination aufgrund eines Verstoßes gegen die 80:20-Regel entzogen wurden.

Flughafenslots sind grundsätzlich weder an eine bestimmte Flugstrecke noch an einen bestimmten Flugzeugtyp gebunden. Sie ermöglichen den Fluggesellschaften eine hohe Flexibilität bei der Strecken-, Flugplan- und Kapazitätsplanung unter Beachtung der Use-it-or-lose-it-Regel. Des Weiteren ist es den Fluggesellschaften erlaubt, Slots untereinander zu tauschen. Demgegenüber ist den Fluggesellschaften der entgeltliche Sekundärhandel mit Slots, also ihr Weiterverkauf an eine andere Fluggesellschaft, in den meisten Jurisdiktionen grundsätzlich untersagt. Eine Ausnahme bilden seit 1986 Level-3-Flughäfen in den USA, wo gemäß der einschlägigen FAA-Regeln den Fluggesellschaften der sekundäre Slothandel gestattet ist (Starkie, 1994). Allerdings hat die FAA das Recht, Slots jederzeit ohne Kompensation zu entziehen. Daneben erklärte 1999 der britische High Court (Merckx, 1999) den entgeltlichen Slothandel zwischen Fluggesellschaften an britischen Flughäfen für rechtskonform mit den Bestimmungen der Verordnung (EWG) Nr. 95/93, die den entgeltlichen Slothandel zwischen Fluggesellschaften weder explizit erlaubt noch explizit verbietet. Er legalisierte damals eine langjährige Praxis, die sich durch Kombination eines formal legalen unentgeltlichen Tauschs zugewiesener Slots mit zugleich hohen Seitenzahlungen insbesondere an den kapazitätsbeschränkten Londoner Flughäfen Heathrow und Gatwick herausgebildet hatte. 2015 führte die chinesische Luftbehörde Civil Aviation Administration of China (CAAC) im Rahmen des „Pilot Reform Program for Market-based Allocation of Time Slot Resources“ eine Slotauktion am Guangzhou Baiyun International Airport (CAN) für Inlandsflüge durch (CAAC, 2016a). Auch am Flughafen Shanghai Pudong (PVG) wurde ein neues Zuteilungsverfahren für sieben Slotpaare erprobt, nämlich eine Lotterie mit Nutzungsgebühr (CAAC, 2016b). An beiden Flughäfen war geplant, 50 % der durch die damals anstehende Inbetriebnahme neuer Pisten geschaffenen zusätzlichen Slots über die genannten Verfahren zuzuteilen (China Daily, 2015). Von der IATA wurde dies als „beunruhigender Präzedenzfall“ kritisiert, der das bewährte WASG-System weltweit langfristig unterminieren könne (Routes, 2016). Am 1.4.2018 führte die CAAC gleichwohl ein neues Slotvergabesystem ein, das neben der Übernahme der IATA-Flughafenklassifikation auch Regelungen für einen Sekundärmarkt für Slots beinhaltet (CAAC, 2018).

## Sonderregeln der EU während der Coronapandemie

In der EU wurde die Use-it-or-lose-Regel zunächst vom 1.3.2020 bis zum 24.10.2020 durch eine Änderung der Verordnung 95/93 vollständig aufgehoben (IATA, 2022c). Am 14.10.2020 wurde deren Aussetzung – der Slot Waiver – bis zum Ende der Winterflugplanperiode am 27.3.2021 verlängert. Am 16.2.2021 wurde eine modifizierte Use-it-or-lose-it-Regel beschlossen, die den Fluggesellschaften für die Sommerflugplanperiode eine Mindestnutzung ihrer Slots von 50 % vorschrieb. Diese Neuregelung wurde am 23.7.2021 für die Dauer der Winterflugplanperiode bis zum 27.3.2022 ausgedehnt. Für die Sommerflugplanperiode vom 28.3.2022 bis zum 29.10.2022 wurde die Mindestnutzungsrate von der EU Kommission am 15.12.2021 schließlich auf 64 % angehoben. Allerdings ist ein Unterschreiten des Schwellenwerts beim Vorliegen „begründeter Ausnahmen der Nichtnutzung“ (JNUS) wie insbesondere pandemiebedingter Reise- oder Quarantänebeschränkungen gemäß dem am 16.12.2021 entsprechend ergänzten Art. 10 der EU-Verordnung 95/93 ohne Slotentzug zulässig.

Offizielles Ziel ist die Vermeidung betriebswirtschaftlich unrentabler und ökologisch fragwürdiger Leerflüge, die von den Fluggesellschaften lediglich durchgeführt worden wären, um dem Entzug ihnen zugewiesener Slots durch die Flugplankoordination zu entgehen. Ökonomisch handelt es sich faktisch um einen Bestandsschutz der an einem als kapazitätsbeschränkt eingestuften Flughafen aktiven Fluggesellschaften, deren ungenutzte Slots für die Dauer des Waivers vor dem Entzug geschützt sind. Entsprechend umstritten ist dieses Instrument unter den Fluggesellschaften. Während der Low Cost Carrier Wizz Air bereits im Sommer 2020 ein Ende der Ausnahmeregelungen forderte (Wizz Air, 2020), beklagte sich der Vorstandsvorsitzende der Lufthansa, Carsten Spohr, in einem Zeitungsinterview kürzlich über 18.000 erzwungene angebliche Leerflüge[3] während der Wintermonate zur Sicherung von Slots (Brankovic und Scherff, 2021).[4]

## Ökonomische Bewertung der Slotvergabepraxis

Als unverzichtbarer Input für die Durchführung eines Fluges stellt die Slotvergabe nach Großvaterrechten eine besonders an den am stärksten frequentierten Verkehrsflughäfen für Newcomer kaum überwindliche Markteintrittsbarrieren dar, die es den Incumbents prinzipiell erlaubt, höhere Ticketpreise und Frachtraten am Markt durchzusetzen. Dies gilt immer dann, wenn die Kapazität des Start- und Landebahnsystems der betroffenen Flughäfen nicht kurzfristig erweitert werden kann oder falls bei einem Nachfrageeinbruch wie infolge der Coronapandemie aufgrund der deutlichen Absenkung der Mindestnutzungswerte durch Slot Waiver ungenutzte Slots nicht entzogen und an interessierte Newcomer realloziert werden dürfen.

Zudem ist zu berücksichtigen, dass der Zugriff auf ein einziges Slotpaar (d. h. für einen Start und eine Landung) für einen wettbewerblich relevanten Marktzutritt in der Regel unzureichend ist. Entscheidend für die Konkurrenzfähigkeit des Angebots des Newcomers gegenüber dem dominanten Incumbent sind die genauen Abflug- und Landezeiten sowie die Möglichkeit, eine den Bedürfnissen der Nachfrager entsprechend hohe Flugfrequenz aufzulegen. Beispielweise müsste der Newcomer auf einer häufig beflogenen Strecke wie etwa Berlin-Frankfurt ebenfalls mehrere tägliche Flugpaare zu attraktiven Zeiten offerieren können, was dessen Slotbedarf substanziell erhöht.

Eine weitere Facette der wettbewerbspolitischen Problematik der Slotvergaberegeln sind die Anreize für strategisches Horten historischer Slots, die entweder nicht (mehr) benötigt werden oder nicht (mehr) betriebswirtschaftlich sinnvoll nutzbar sind. Diese Praxis, mit der der Marktzutritt von Konkurrenz insbesondere an den Level-3-Flughäfen effektiv verhindert werden kann, wird auch als Slot Sitting bzw. Slot Squatting bezeichnet. Es kann immer dann auftreten, wenn

- ein Flug mangels Nachfrage zwar dauerhaft nicht (mehr) kostendeckend durchgeführt werden kann (bzw. bei Umsteigeverbindungen über das Drehkreuz der Fluggesellschaften keinen positiven Netzbeitrag – also einen positiven Deckungsbeitrag zu den Kosten des Gesamtstreckennetzes – liefert), die Fluggesellschaft den Wert des Slots als rentengenerierende Markteintrittsschranke aber höher veranschlagt als die durch dessen Nutzung anfallenden Verluste, oder
- der Slotinhaber größere Flugzeuge durch kleinere ersetzt, obwohl dies eine ökonomisch ineffiziente Fehlallokation knapper Flughafeninfrastrukturen darstellt.

Nicht übersehen werden darf, dass die Slotvergaberegeln ohnehin nur äußerst geringe ökonomische Anreize bieten, einmal erteilte Slots zurückzugeben. So ist die Chance, bei Bedarf kurz- bis mittelfristig neue, zeitlich passende Slots an kapazitätsbeschränkten Flughäfen zu erhalten, in der Regel äußerst gering. Somit können von den Slotvergaberegeln auch potenziell konzentrationsfördernde Wirkungen ausgehen. Dies ist immer dann der Fall, wenn das vorhandene Slotportfolio an kapazitätsbeschränkten Flughäfen eine Fluggesellschaft zu einem attraktiven Übernahmeobjekt werden lässt und durch einen Zusammenschluss eine bereits bestehende marktbeherrschende Stellung verstärkt oder eine neue marktbeherrschende Stellung begründet würde. Sowohl in den USA als auch in der EU werden im Rahmen der Fusionskontrolle sowie bei kartellrechtlichen Prüfungen horizontaler Vereinbarungen zwischen Fluggesellschaften regelmäßig behördliche Auflagen erteilt, die die Weitergabe einer bestimmten Zahl von Slots an interessierte Wettbewerbende als zwingende Voraussetzung für eine Genehmigung vorsehen (gegebenenfalls ergänzt um die Aufgabe von einzelnen Flugstrecken oder Frequenzen zugunsten von Newcomern).

Neben dem Zuteilungsverfahren können die Incumbents aber schließlich auch das Verfahren zur Rückgabe nicht benötigter Slots als strategische Markteintrittsbarriere missbrauchen. Dies ist möglicherweise immer dann der Fall, wenn der Stichtag für die Rückgabe eines erteilten Slots an die Flugplankoordination zeitlich zu kurz vor Beginn der Flugplanperiode liegt. Die Neuvergabe des Slots – außer für eine niedrigfrequente Ad-hoc-Nutzung – wird aufgrund des erforderlichen zeitlichen Vorlaufs bei der Flugplanerstellung dadurch zumindest erheblich erschwert.

Hinsichtlich der Betriebsabläufe begünstigt das Slotvergabesystem auf mehreren Ebenen einseitig die Fluggesellschaften gegenüber den Flughäfen. So werden Slots den Fluggesellschaften von der zuständigen Flugplankoordination grundsätzlich unentgeltlich zugeteilt. Doch nur an

3 Die von Spohr gewählte Formulierung Leerflüge ist irreführend, da diese in aller Regel nicht leer durchgeführt werden – d. h. ohne Passagiere und/oder Fracht –, sondern nur eine schlechte Buchungslage aufweisen.

4 Die Praxis temporärer Slot Waiver ist nicht neu. Schon vor der Coronapandemie waren diese wiederholt infolge massiver temporärer Nachfrageeinbrüche ausgesprochen worden, etwa nach den Terroranschlägen am 11.9.2001, während der SARS-Pandemie (2002 bis 2003), im Zuge der globalen Finanzkrise 2008 sowie nach dem Ausbruch des isländischen Vulkans Eyjafjallajökull am 14.4.2010.

unausgelasteten Flughäfen (Level 1) ist ihr ökonomischer Wert auch null. Herrscht an einem Flughafen eine temporäre oder permanente Übernachfrage nach Slots, müsste um einer effizienten Allokation willen jeder Slot zum markträumenden Knappheitspreis zugewiesen werden – was bis auf die genannten Ausnahmen (teilweise in den USA und Großbritannien) weltweit nicht der Fall ist. Der Slotbestand einer Fluggesellschaft an kapazitätsbeschränkten Verkehrsflughäfen begründet damit die Umleitung ökonomischer Knappheitsrenten von den Flughäfen als den Eigentümern dieser Infrastrukturen zu den dort aktiven Fluggesellschaften und damit implizit eine Subventionierung Letzterer durch Erstere. Der wirtschaftliche Wert der historischen Slots an den hochfrequentierten Flughäfen ist erheblich,[5] lässt sich aber ohne Slothandel nur indirekt als immaterieller Vermögensgegenstand anhand der Pflichtangaben der Fluggesellschaften im Rahmen der externen Rechnungslegung näherungsweise bestimmen (Olbrich, Brosel und Hasslinger, 2009); Olbrich und Hasslinger, 2010); beispielsweise bilanzierte Air Berlin 2015 176 Mio. Euro für nicht näher bezeichnete „Landerechte“ (Kuhn, 2016). Sie spielen darüber hinaus auch eine bedeutende Rolle als Sicherheiten bei der Kapital- und Kreditaufnahme von Fluggesellschaften, insbesondere in den USA und Großbritannien.

Darüber hinaus genießen die Fluggesellschaften aufgrund ihrer Bestände an historischen Slots umfassende Flexibilität bei deren Nutzung und ein hohes Maß an Planungssicherheit bei der Flugplanerstellung und der Organisation ihrer betrieblichen Abläufe. Dies gilt insbesondere für den hochkomplexen Flugbetrieb an den Drehkreuzen. Für die Flughafenbetreiber bedeutet die im Normalfall lediglich 80 %ige Mindestnutzungspflicht eines Slots eine im Extremfall 20 %ige Unterauslastung der von ihnen bereitgestellten luftseitigen Infrastruktur sowie die daraus resultierenden Mindereinnahmen aus der Abfertigung von Flugzeugen, Passagieren und Fracht sowie im Non-Aviation-Bereich. In Krisensituationen wie der Coronapandemie hindern die Slotvergaberegeln die Flughafenbetreiber darüber hinaus an der Neukundenakquise, da expansionswilligen Newcomern der Zugang zu den benötigten Slots trotz aktuell stark unterausgelasteter Kapazitäten verwehrt bleibt.

Betrachtet man Slots aus der Perspektive der Theorie der Verfügungsrechte (Eggertson, 1990; Hart, 1995) ist festzuhalten, dass in der einschlägigen juristischen Literatur deren Rechtsnatur bis heute nicht eindeutig und vollständig geklärt ist. Die für die EU einschlägige Verordnung (EWG) Nr. 95/93 definiert Slots in Artikel 2 als „die von einem Koordinator gemäß dieser Verordnung gegebene Erlaubnis, die für den Betrieb eines Luftverkehrsdienstes erforderliche Flughafeninfrastruktur eines koordinierten Flughafens an einem bestimmten Tag und zu einer bestimmten Uhrzeit, die von einem Koordinator nach dieser Verordnung zugewiesen wurden, in vollem Umfang zum Starten oder Landen zu nutzen“. Gemäß der inhaltlich sehr ähnlichen Definition der US-Flugaufsichtsbehörde FAA handelt es sich um die “authorization to either take-off or land at a particular airport on a particular day during a specified time period … to prevent repeated delays [at extremely busy airports] that result from too many flights trying to take off or land at the same time” (FAA, 2022). Slots werden den Fluggesellschaften von staatlichen Stellen somit lediglich als zeitlich klar definierte, befristete und exklusive Zugangs- und Nutzungsrechte für luftseitige Flughafeninfrastrukturen, insbesondere Start- und Landebahnen, zur Verfügung gestellt. Die beiden Definitionen spiegeln das gesetzgeberische Dilemma wider, den Fluggesellschaften (oder Dritten) einerseits nicht das alleinige Eigentum an Slots zu übertragen, andererseits jedoch ihren Inhabern Rechtssicherheit bezüglich der ihnen zugewiesenen Nutzungsrechte bieten zu wollen.

Ökonomisch betrachtet verfügen die Fluggesellschaften über hochspezifizierte Eigentumsrechte an den ihnen zugewiesenen Slots. Diese umfassen im Sinn der Property-Rights-Theorie nicht nur das infolge des Grandfathering langfristige exklusive Nutzungsrecht (Usus). Auch sämtliche (positiven wie negativen) Nettoerträge aus der Nutzung (Usus fructus) entfallen auf sie. Das zulässige „Retiming“ sowie der ebenfalls legale Naturaltausch von Slots ist gleichbedeutend mit dem Recht zur Veränderung (Abusus). In Jurisdiktionen, in denen der entgeltliche Sekundärhandel zulässig ist, tritt noch das Recht zur Veräußerung (Ius abutendi) hinzu. Verdünnt werden die Eigentumsrechte nur durch die Pflicht zur Einhaltung der Use-it-or-lose-it-Regel. Eine vollständige Entwertung kann nur durch den Entzug von Slots durch die Flugplankoordination oder bei Insolvenz der Fluggesellschaft erfolgen (sofern deren Slotbestand nicht Teil der Konkursmasse ist). Eine partielle Entwertung ist denkbar:

- *Erstens* bei einer spürbaren Erweiterung der Flughafenkapazitäten, sofern diese in substanziellem Umfang Markteintritte konkurrierender Anbieter nach sich zöge.
- *Zweitens* durch effektiven Wettbewerb zwischen ungleich ausgelasteten Flughäfen innerhalb eines überlappenden Einzugsgebiets (Catchment Area), die um substituierbare Verkehrsströme konkurrieren, wie z. B. die Drehkreuze unterschiedlicher Airlines bzw. Allianzen um Umsteigeverkehre auf Mittel- und Langstreckenverbindungen.

## Mögliche Reform des Slotvergabesystems

Die Schwächen des Slotvergabesystems werden bereits seit Jahrzehnten ebenso regelmäßig diskutiert, wie mögli-

5 Beispielsweise erwarb Oman Air im Februar 2016 für den Flughafen London Heathrow ein Slotpaar (d. h. für eine Landung und einen Start) von Air France für 75 Mio. US-$ (Borders, Huff und Nussbaum, 2020).

che Reformvorschläge propagiert werden. Dabei werden in der ökonomischen Diskussion zwei alternative Vergabeverfahren zur Steigerung der Effizienz bei der Nutzung von Slots eindeutig bevorzugt: regelmäßige Slotauktionen für die Erstzuteilung von Slots und/oder ein ergänzender kommerzieller Sekundärmarkt zwischen den Fluggesellschaften für deren Reallokation (Levine, 1987; NERA Economic Consulting, 2004; Czerny et al., 2008; Pellegrini et al., 2012; Pertuiset und Santos, 2014; Davies, 2019). Dabei wird implizit vorausgesetzt, dass die Erstzuteilung von Slots durch den Staat – vertreten durch die nationale Flugplankoordination oder die Flugaufsichtsbehörde – erfolgt. Offen bleibt, wem die Auktionserlöse aus der Erstzuteilung an die Fluggesellschaften zufließen sollen. Grundsätzlich können dies sein:

- Der Staat als Bereitsteller der Slots, wobei der erzielte Versteigerungserlös den Charakter eines Nutzungsentgelts hätte, oder
- der Flughafenbetreiber als Bereitsteller und Finanzier der zugrunde liegenden und über die Slotvergabe rationierten Infrastrukturen.

Die auf dem nachgelagerten Sekundärmarkt erzielten Erlöse aus dem Verkauf oder Verleasen von Slots würden derjenigen Fluggesellschaft zufließen, die sich im Rahmen der Erstzuteilung ein Zeitnischennutzungsrecht ersteigern konnte.

### Szenario I

Somit ergeben sich verschiedene Basisszenarien mit unterschiedlichen Wettbewerbseffekten. In Szenario I würde die Erstausstattung der Fluggesellschaften mit Slots weiterhin kostenlos und unter Beibehaltung des über das IATA-System von den Fluggesellschaften faktisch selbst geschaffenen (Gewohnheitsrechts-)Instituts historischer Slots erfolgen. Zugleich würde ein kommerzieller Sekundärmarkt zwischen den Fluggesellschaften eingeführt, um die Reallokation von Slots zu erleichtern. Dieses Arrangement würde jedoch die bisherigen Inhaber von Slots gegenüber Newcomern massiv bevorzugen. Neben den hier zugunsten der Slotinhaber generierten bilanzrelevanten Windfallprofits würde auch deren Marktmacht an den hochfrequentierten Verkehrsflughäfen mutmaßlich weiter zementiert. So könnten Newcomer den Markteintritt zu diesen Flughäfen im Gegensatz zu den Incumbents entweder nur zu deutlich höheren Kosten erreichen (durch den Ankauf von Slots von den Incumbents – denen ihre Slots ursprünglich in der Regel kostenlos zugewiesen worden waren). Alternativ könnte den Newcomern der Markteintritt effektiv verwehrt bleiben, sofern die Slotinhaber durch „Slot Sitting" eine Strategie der Marktschließung durch Behinderungsmissbrauch in Form einer „Vertical Upstream Foreclosure" verfolgen. Somit ließe sich keine knappheitsorientierte Allokation und damit auch keine effiziente Nutzung der verfügbaren Slots gewährleisten.

### Szenario II

Szenario II ist durch die Kombination einer von der zuständigen staatlichen Stelle vor jeder Flugplanperiode abgehaltenen Auktion zur Erstzuteilung der Slots an Fluggesellschaften mit einem kommerziellen Sekundärhandel zwischen den Fluggesellschaften charakterisiert. In diesem Szenario wären nicht nur historische Slots abgeschafft. Auch die Notwendigkeit einer Use-it-or-lose-it-Regel bestünde nicht mehr, da alle Slotinhaber im Gegensatz zum Status quo ein starkes betriebswirtschaftliches Interesse an der bestmöglichen Nutzung der von ihren nur temporär zur Verfügung stehenden Zeitnischen hätten. Nicht nur die bisher existenten wettbewerbsbeschränkenden Hortungs- bzw. Slot-Sitting-Anreize wären deutlich geringer als bei Fortdauer des Status quo. Darüber hinaus ist eine effizientere Nutzung der Infrastruktur durch deren Allokation zu Knappheitspreisen und den Wegfall der Use-it-or-lose-it-Regel zu erwarten.

### Szenario III

Denkbar wäre ein weiteres Szenario III, das nach dem Kenntnisstand der Verfasser in der Literatur bislang nicht diskutiert wurde. Es beruht weitgehend auf Szenario II, da es wie dieses die Kombination aus zeitlich befristeter auktionsbasierter Erstzuteilung und kommerziellem Sekundärhandel zwischen den Fluggesellschaften vorsieht. Der fundamentale Unterschied besteht allerdings darin, dass zunächst sämtliche Flughafenslots den Flughäfen übertragen werden, d.h. den Eigentümern und Betreibern der relevanten knappen Infrastruktur, deren Nutzung durch Slots rationiert werden muss. Es handelt sich also um eine fundamentale Neuordnung des institutionellen Arrangements, da die mit den Slots verknüpften Eigentumsrechte, insbesondere die Disposition und Nutzung der Start- und Landerechte, der Sphäre der Infrastrukturbetreiber zugeordnet werden. Auch hinsichtlich des institutionellen Arrangements zur Festlegung der Koordinationseckwerte werden Änderungen vorgeschlagen. Diese sollten im Szenario III von den Flughafenbetreibern selbst in Kooperation mit der Flugsicherung, aber ohne Mitbestimmung Dritter festgelegt werden, um eine effiziente Nutzung der knappen Ressource Flughafenkapazität sicherstellen zu können. In einem solchen Szenario entspricht das betriebswirtschaftliche Interesse des Flughafens an einer bestmöglichen Nutzung seiner Infrastrukturen dem wettbewerbspolitischen Interesse an einer Reduktion der aus der bisherigen Slotvergabepraxis resultierenden Markteintrittsbarrieren. Zu erwarten ist ein höherer Nutzungsgrad der verfügbaren Slots, da der Flughafenbetreiber nunmehr in der Lage wäre, während einer Flugplanperiode frei gewordene Slots zügig für deren Restdauer zu reallozieren und so die Auslastung seiner luft- und bodenseitigen Infrastrukturen zu optimieren. Das bisherige System, einschließlich der derzeitigen Slot Waiver, verlagert das wirtschaftliche Risiko einer starken Reduzierung der Flugaktivitäten einer oder mehrerer Fluggesellschaften einseitig auf den Flughafenbetreiber, da diesem die Möglichkeit nicht offensteht, die aus rechtlichen Gründen zurückgehaltenen Infrastrukturkapazitäten an interessierte Newcomer weiterzugeben und dadurch Neugeschäft zu generieren. Des Weiteren stünde den Flughafenbetreibern eine nicht nur nutzungs-, sondern auch knappheitsbasierte zusätzliche Finanzierungsquelle für Erhaltungs- und Ausbauinvestitionen der luftseitigen Infrastrukturen zur Verfügung.

## Diskussion der Szenarien

Von den großen, auf Umsteigeverkehre über ihre jeweiligen Drehkreuze spezialisierten Fluggesellschaften wird eine marktbasierte Slotvergabe und vor allem die Abschaffung historischer Slots regelmäßig abgelehnt. Diese stünde der erforderlichen langfristigen Netzplanung entgegen bzw. würde deren Komplexität substanziell erhöhen. Überzeugend ist dieses Argument nicht, da innerhalb der bestehenden Verbünde wie der Lufthansa Group sowie der großen Allianzen (Star Alliance, Skyteam und Oneworld) regelmäßig Streckennetze optimiert und Verkehrsflüsse flexibel über alternative Drehkreuze geleitet werden. Dahinter verbirgt sich der aus Sicht der etablierten Fluggesellschaften mit umfangreichen Slotportfolios an kapazitätsbeschränkten Flughäfen betriebswirtschaftlich nachvollziehbare Wunsch, sich die ihnen durch den Status quo garantierten Renten weiterhin zu sichern. Hieraus resultierende Rent-Seeking-Aktivitäten führen aber zu ökonomischen Ineffizienzen und Wohlfahrtsverlusten. Es bedeutet insbesondere eine Fortschreibung der ineffizienten indirekten Subventionierung des Infrastrukturzugangs zugunsten der Inhaber historischer Slots.

Die Umsetzung von Szenario III wirft ebenfalls wettbewerbspolitische Fragen auf, etwa nach der Marktmacht der Flughäfen gegenüber den Fluggesellschaften und damit nach der Wahrscheinlichkeit missbräuchlich überhöhter Preise bei der Slotzuteilung. Marktmacht kapazitätsbeschränkter Flughäfen ist bei regionaler Alleinstellung grundsätzlich anzunehmen. Einen möglichen Missbrauch dieser Marktmacht zu unterbinden, bliebe eine Aufgabe der Wettbewerbspolitik, die mit der Missbrauchsaufsicht über ein geeignetes Eingriffsinstrumentarium verfügt. Allerdings erscheint das Risiko eines solchen Missbrauchs gering. Die Erstzuteilung erfolgt über eine Versteigerung, und die daran teilnehmenden Fluggesellschaften können die Slots realistisch bewerten; überzogene Mindestgebote des Flughafenbetreibers sind daher mit dem Risiko behaftet, Slots nicht vergeben zu können und Einnahmen zu verlieren. Wie bei jedem Systemwechsel sind jedoch Übergangsprobleme denkbar. Darüber hinaus sollte nicht übersehen werden, dass gerade auch die besonders kapazitätsbeschränkten Großflughäfen wie Frankfurt oder München bei Umsteigeverkehren auf der Langstrecke und längeren Mittelstrecken in einem intensiven Wettbewerb zu ausländischen Drehkreuzen stehen, was deren Missbrauchsspielräume effektiv begrenzen sollte. Bei kürzeren Verbindungen wären in diesem Zusammenhang die Konkurrenz zu anderen Flughäfen mit überlappendem Einzugsgebiet sowie Art und Umfang des intermodalen Wettbewerbs, etwa durch den Schienenverkehr, von Relevanz.

Nicht auszuschließen wäre auch eine Kollusion zwischen dem Flughafenbetreiber und den an ihrem Standort dominanten Fluggesellschaften oder Allianzen zum Nachteil von Newcomern. In Szenario III wäre dies allerdings weniger ein durch die Erstauktion von Slots oder deren Sekundärhandel hervorgerufenes Problem. So könnte sich eine Eintrittsbarriere für Newcomer aus dem fehlenden oder unzureichenden Zugang zu den für die Abfertigung der Flüge benötigten komplementären bodenseitigen Infrastrukturen des Flughafens ergeben, sofern diese vom Flughafenbetreiber langfristig an eine Fluggesellschaft oder Allianz verpachtet wurden. In diesem Fall wäre der Newcomer trotz der Zuteilung von Slots nicht in der Lage, Flüge durchzuführen oder bei Ersteigerung zusätzlicher Slots zu expandieren.

## Fazit und Ausblick

Infolge der äußerst dynamischen Entwicklung des zivilen Luftverkehrs seit Ende des Zweiten Weltkriegs nahm die Zahl der kapazitätsbeschränkten Flughäfen in allen bedeutenden Verkehrsregionen, insbesondere aber in Europa, wo sich die weltweit mit Abstand höchste Zahl von Level-2- und Level-3-Flughäfen befindet, deutlich zu. Bei der erwarteten Erholung des Luftverkehrsmarktes nach Überwindung der Coronapandemie und den bekannten Problemen des Flughafenaus- und -neubaus in vielen EU-Mitgliedstaaten ist mittelfristig mit einer weiteren Verschärfung der Infrastrukturknappheit zu rechnen. Der noch anhaltende Einbruch der Passagierzahlen infolge der vielerorts geltenden Reisebeschränkungen und Quarantänevorschriften sowie der Zurückhaltung vieler Nachfragender bietet eine historisch einmalige Chance für eine radikale Reform des bisherigen Systems der Vergabe von Flughafenslots. So mussten viele Fluggesellschaften ihre Flugpläne, d.h. die Zahl ihrer Destinationen sowie der angebotenen Frequenzen, erheblich ausdünnen. Die aktuelle Situation böte Newcomern somit grundsätzlich weitreichende Möglichkeiten zum Markteintritt und Expansion an den vor der Pandemie hochfrequentierten und entsprechend slot-beschränkten Großflughäfen. Die von den zuständigen Behörden weltweit erlassenen Slot Waiver verhindern dies jedoch. Sie sollten daher aufgehoben werden, gerade auch um den pandemiebedingten Nachfrageeinbruch zur Dekonzentration der vielerorts durch marktbeherrschende Fluggesellschaften und ihre Allianzen vermachteten Luftverkehrsmärkte zu nutzen.
